# Using deep learning models to analyze the cerebral edema complication caused by radiotherapy in patients with intracranial tumor

**DOI:** 10.1038/s41598-022-05455-w

**Published:** 2022-01-28

**Authors:** Pei-Ju Chao, Liyun Chang, Chen-Lin Kang, Chin-Hsueh Lin, Chin-Shiuh Shieh, Jia-Ming Wu, Chin-Dar Tseng, I-Hsing Tsai, Hsuan-Chih Hsu, Yu-Jie Huang, Tsair-Fwu Lee

**Affiliations:** 1 Department of Electronics Engineering, National Kaohsiung University of Science and Technology, Kaohsiung, 80778 Taiwan, ROC; 2 Medical Physics and Informatics Laboratory of Electronics Engineering, National Kaohsiung University of Science and Technology, Kaohsiung, 80778 Taiwan, ROC; 3 Department of Radiation Oncology, Kaohsiung Chang Gung Memorial Hospital and Chang Gung University College of Medicine, Kaohsiung, 83342 Taiwan, ROC; 4 Department of Medical Imaging and Radiological Sciences, I-Shou University, Kaohsiung, 82445 Taiwan, ROC; 5 Department of Biomedicine Engineering, Chengde Medical University, Chengde Hebei, 067000 China; 6 PhD Program in Biomedical Engineering, Kaohsiung Medical University, Kaohsiung, 80708 Taiwan, ROC

**Keywords:** Cancer, Engineering, Optics and photonics

## Abstract

Using deep learning models to analyze patients with intracranial tumors, to study the image segmentation and standard results by clinical depiction complications of cerebral edema after receiving radiotherapy. In this study, patients with intracranial tumors receiving computer knife (CyberKnife M6) stereotactic radiosurgery were followed using the treatment planning system (MultiPlan 5.1.3) to obtain before-treatment and four-month follow-up images of patients. The TensorFlow platform was used as the core architecture for training neural networks. Supervised learning was used to build labels for the cerebral edema dataset by using Mask region-based convolutional neural networks (R-CNN), and region growing algorithms. The three evaluation coefficients DICE, Jaccard (intersection over union, IoU), and volumetric overlap error (VOE) were used to analyze and calculate the algorithms in the image collection for cerebral edema image segmentation and the standard as described by the oncologists. When DICE and IoU indices were 1, and the VOE index was 0, the results were identical to those described by the clinician.The study found using the Mask R-CNN model in the segmentation of cerebral edema, the DICE index was 0.88, the IoU index was 0.79, and the VOE index was 2.0. The DICE, IoU, and VOE indices using region growing were 0.77, 0.64, and 3.2, respectively. Using the evaluated index, the Mask R-CNN model had the best segmentation effect. This method can be implemented in the clinical workflow in the future to achieve good complication segmentation and provide clinical evaluation and guidance suggestions.

## Introduction

Radiotherapy is one of the techniques of clinical tumor treatment. In patients with intracranial tumors, if the tumor is located in the deep part, it will be difficult to use surgical techniques to remove the tumor. Therefore, radiotherapy plays a very important role in tumor treatment. Tumor treatment has gotten more accurate by using the image diagnosis^1^. Common clinical imaging methods for intracranial tumors include computed tomography (CT), magnetic resonance imaging (MRI), Angiography, and other medical instruments.

Clinically, high-precision stereotactic radiosurgery (SRS) technology is used to diagnose brain tumor patients^2–4^. The principle of clinical treatment is using an accurate tumor radiation dose to improve the control rate. The dose in surrounding normal tissues is reduced to decrease side effects.

In SRS, the radiation irradiates the human body with multiple angles and a large dose to kill tumor cells like a scalpel, which can improve the tumor control rate^5,6^. However, the relatively high-dose relationship may damage the normal tissues surrounding the tumor, and cause early complications of radiation cerebral edema^7^, which will affect the patient’s therapeutic efficacy and quality of life, so the evaluation of complications and treatment of the disease are relatively important^8^.

It is necessary to accurately delineate the normal organs at risk (OAR) on the patient’s CT images before designing a radiotherapy plan. Auto segmentation contours (ASC) are commonly used in the treatment planning system (TPS) in current medical technology, such as RayStation, MIM vista, and MIRADA, which can accurately outline the patient’s OAR contour area^9^. This can manually and automatically assist clinical physicists in describing these organs. Medical physicists will assist oncologists in drawing up patient treatment plans in the treatment planning system software^10–12^, MultiPlan. In patients with intracranial tumors, there is no automatic description software of the complications of cerebral edema after stereotactic radiotherapy. Cerebral edema is not conducive to penetration due to the similar density of intracranial tissues and the uncertainty of location and size. The gray gradient calculation method obtains the cerebral edema contour so that this automatically drawn software cannot effectively outline the complication area. There is no complete software basis construction and description process for cerebral edema complications caused by radiotherapy. The current clinical description of complications must rely on the professional manual description of the clinical radiation oncologist, which usually involves multiple fragments. The result of this process depends on the oncologist’s experience and subjective judgment. Different oncologists will also lead to different areas of cerebral edema^13,14^. Therefore, we investigate the introduction of artificial intelligence (AI) for cerebral edema images using deep learning segmentation (DLS) and clinicians’ description standard analysis^15,16^. This study hopes to establish a good automatic description of cerebral edema complications software, so that clinicians have a reference basis on the auxiliary definition in judging cerebral edema complications. The study exports the clinical cerebral edema imaging data from the treatment planning system to a personal computer. Based on the above reasons, this study evaluates two different types of image segmentation methods: (1) area growth, and (2) mask region-based convolutional neural networks (Mask R-CNN). The analysis is most similar to the clinician’s standard for describing a cerebral edema area.

The recent main methods in the field of image segmentation and deep learning applications are referred to for this study. Convolutional neural networks (CNNs) have excellent accuracy in image segmentation. This technology has been a breakthrough in computer vision processes. However, In the medical field, labeling medical images require a large amount of professional discrimination and is time-consuming by clinicians, so it is a challenge to obtain accurate and large amounts of labeled data sets.

**Figure 1:**
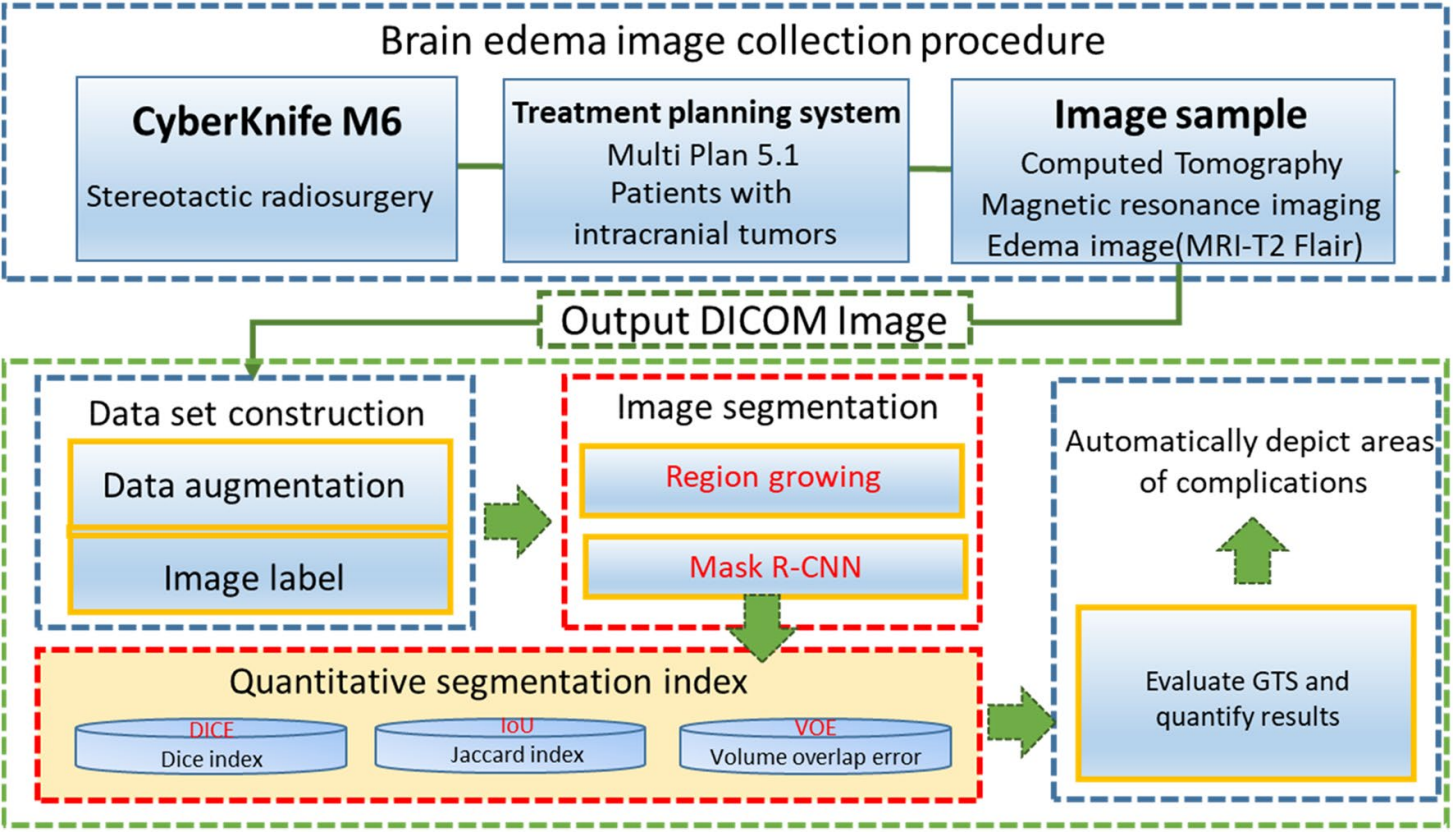
Flow chart of this study. *MRI* magnetic resonance imaging, *DICOM* digital imaging and communications in medicine, *R-CNN* region-based convolutional neural networks, *IoU* intersection over union, *VOE* volumetric overlap error, *GTS* ground truth segmentation. Image source statement: This image was created using Microsoft PowerPoint 2016. Software URL: https://www.office.com/.

When the available data set size is limited, data enhancement technology can improve model performance and solve the problem of insufficient training data for medical imaging models. Data enhancement technology is also very important for medical applications, such as organ segmentation and complication detection^17,18^. Figure 1 shows a flow chart of this research.

## Materials and methods

### Image acquisition

This study was approved by the institutional review boards of Chang Gung Memorial Hospital (201900565B0), and all experiments were performed in accordance with relevant guidelines and regulations. The images of cerebral edema caused by patients with intracranial tumors that received radiotherapy for four months were provided by the radiation oncologist of Chang Gung Memorial Hospital, Kaohsiung City, Taiwan. The medical physicist exported intracranial tumors patient imaging data from the current treatment planning system software, MultiPlan, including digital medical images: CT, MRI, and cerebral edema imaging area (22 edema images).

The cerebral edema images were not many for patients with four months of clinical treatment. Therefore, bilinear interpolation was performed through MIM vista (MIM Software, Cleveland, OH), and 109 cerebral edema images were obtained after interpolation. The clinician will describe the standard cerebral edema image as the standard result (ground truth segmentation, GTS), and then use data enhancement technology to expand the cerebral edema sample to 700 for model training. The medical image specifications exported by this study are shown in Table 1.

**Table 1:** The medical image specifications.

Image type	Slice thickness (mm)	Number of images	Image resolution	Pixel spacing (mm)
CT	0.625	427	512*512	[0.7422,0.7422]
MRI (T2-Flair, after treatment)	5.000	22	320*320	[0.7188,0.7188]
MRI (T2-Flair, after image fusion)	0.625	109	512*512	[0.7211,0.7211]
MRI (T2-Flair, data enhancement)	0.625	700	512*512	[0.7211,0.7211]

*CT* computed tomography, *MRI* magnetic resonance imaging.

**Figure 2:**
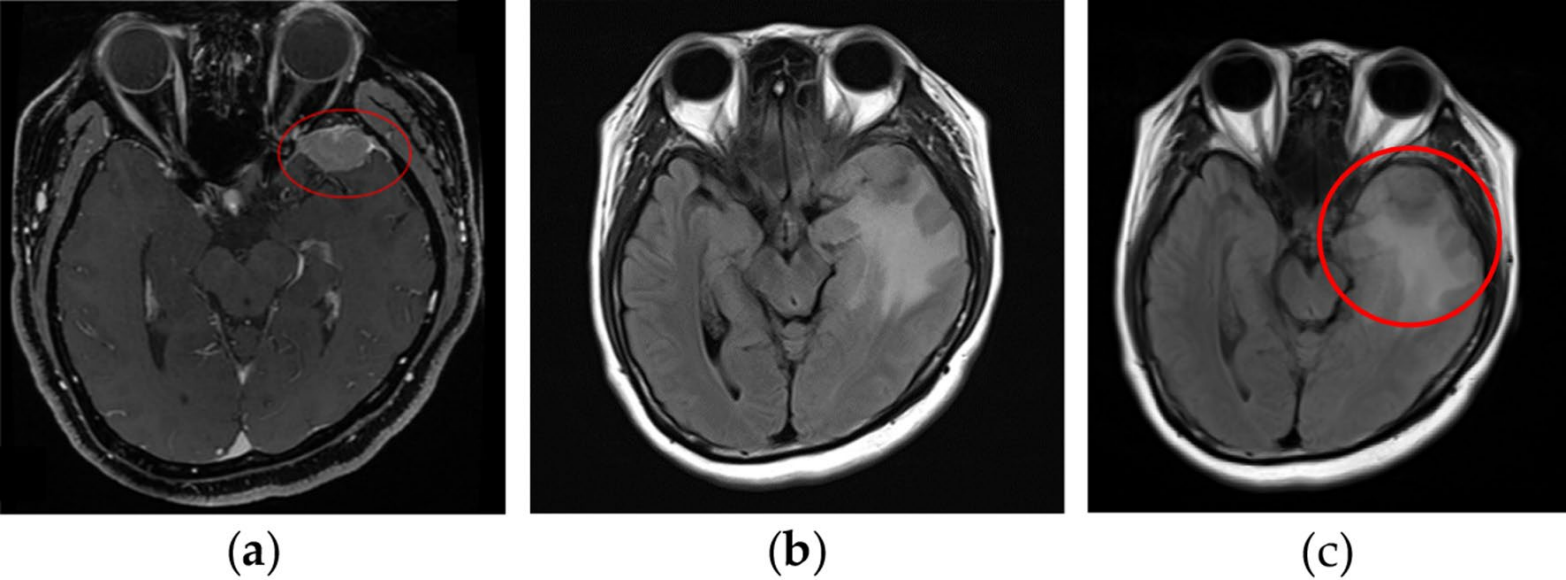
MRI images before treatment and MRI images after image fusion. (**a**) MRI before treatment. (**b**) MRI 4 months after treatment. (**c**) Cerebral edema area with fusion technology. *MRI* magnetic resonance imaging. Image source statement: This image was created using CyberKnife M6 system. Software URL: https://www.accuray.com/resources/accurays-cyberknife-m6-series/.

Clinically, MRI information is provided to the physician to observe the image of the patient’s lesion. Figure 2a shows the MRI before treatment, where the area drawn by the red circle is the tumor area before treatment. Figure 2b is an MRI T2-Flair image taken four months after treatment. The white area is the radioactive cerebral edema area we studied. The original small number of cerebral edemas is interpolated into a new image through MIM vista image fusion technology (109 photos). The red circle in Fig. 2c shows the cerebral edema area.

### Data set construction and annotation

**Figure 3:**
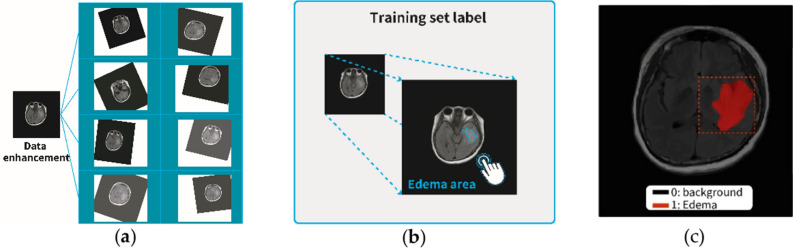
Data set construction and annotation. (**a**) Data enhancement technology. (**b**) Image annotation tool, Labelme. (**c**) Image marked with cerebral edema. Image source statement: This image was created using CyberKnife M6 system. Software URL: https://www.accuray.com/resources/accurays-cyberknife-m6-series/.

The size of the cerebral edema image used in the study is 512 × 512 pixels. To increase the number of cerebral edema image samples, the data enhancement technology was used, as shown in Fig. 3a. The purpose of increasing the number of image samples was to ensure that the image model avoided overfitting and increase prediction accuracy when training on this image set. Seven hundred images were randomly selected for the Mask R-CNN model building. The images of 80 percent were used as the training set, and 20 percent was used as the validation set. After the training was completed, the remaining 100 images were used for testing to evaluate the performance of the trained model.

Labelme is used as an image annotation tool for experimental data to generate a Mask image of cerebral edema. They are shown in Fig. 3b. The reverse loss function in the Mask image calculation model was used as training and model parameters. In addition, the trained segmented Mask area was compared with the standard described by the radiation oncologist. The performance of the model instance segmentation and the result of the second algorithm (region growing) were evaluated. The cerebral edema area of the image is the main category label, and the rest of the area defaults to the background. The image marked with cerebral edema is shown in Fig. 3c.

### Target segmentation of region growing

The region growing algorithm is used as a set seed to select the cerebral edema area. After setting the seed, it is distinguished according to the pixel intensity (grayscale value, color) around the cerebral edema image to make the cerebral edema contour split it out. The cerebral edema image segmentation is shown in Eq. (1), which represents the absolute value of the pixel P and the seed point S. The difference less than 20% between the maximum intensity value max(R) and the minimum intensity value min(R) in the growing region is growth conditions, otherwise stop.

1 $$\left|P-S\right|\le 20\%\left[max\left(R\right)-min\left(R\right)\right]$$

Multiple seeds are set to segment each layer of the cerebral edema regions. The segmentation results of the region growing algorithm may be different from the cerebral edema complications described by the clinician. The reason for the different results of the two segmentation methods is that the clinician will consider whether the damaged cells in the surrounding complication area needs to be evaluated, and the outline range is drawn larger. The region growing algorithm will segment according to the surrounding intensity and will not over-segment other regions with dissimilar intensity.

### Target segmentation model structure of Mask R-CNN

Mask R-CNN is the latest method in the field of target detection. It extends the target detection framework of Faster R-CNN. There is an extra branch at the end of the Faster R-CNN model. The fully connected layer (FC) is used to implement instance segmentation for each output suggestion box, which can simultaneously segment, identify, and locate tasks.

**Figure 4:**
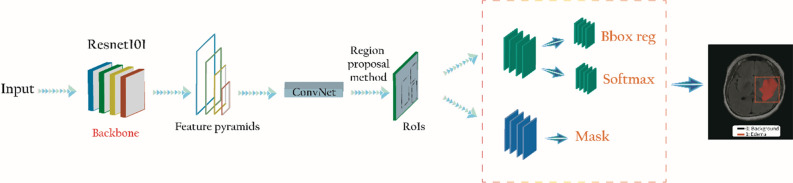
Mask R-CNN network architecture diagram. *RoI* generate a region of interest. Image source statement: this image was created using Microsoft PowerPoint 2016. Software URL: https://www.office.com/.

The backbone network extracts feature maps from the input cerebral edema image through the CNN network. The feature map output by the backbone network is sent to the region proposal network (RPN) to generate a region of interest (RoI). The RoI is mapped to extract the corresponding target features in the shared feature map and output to the FC and full convolutional network (FCN) for target classification and instance segmentation. This process will generate cerebral edema image regions. The Mask R-CNN framework includes three stages, which are shown in Fig. 4.

To define the loss function, the Mask R-CNN uses a multi-object loss function, e.g., as shown in Eq. (2), here L_cls_ is the classification error of the neural network, L_box_ represents the detection error, and L_mask_ represents the segmentation error^19^.

2 $$L={L}_{cls}+{L}_{box}+{L}_{mask}$$

#### Feature extraction and generation of RoIs (ResNet101 + FPN + RPN)

Feature extraction and RoI generation with 50 and 101 layers (ResNet50 /101 + FPN + RPN) create deep neural network models with different depths by designing different weight layers. Currently, AlexNet, VGG, GoogleNet, and deep residual network (ResNet) models are the main models of deep neural networks^20–22^. A deeper network may bring higher accuracy. However, a deeper network reduces the speed of model training and detection. The ResNet structure does not increase the model parameters, which effectively alleviate the difficulty of gradient disappearance and training degradation and improve the convergence performance of the model. Therefore, ResNet was used as the backbone network in this research^23^.

Image feature extraction is based on a shared convolutional layer. Low-level features (such as edges and angles) are obtained through the basic network. However, the category of cerebral edema is high-level features that are extracted at a higher level. To make the detection of small targets particularly effective, cerebral edema image information is extended to the backbone network. The model is designed with the function of feature pyramid networks (FPN) architecture through up-sampling and connection with basic functions so that each layer of the network can be independently predicted.

The convolutional feature map is output from the backbone network, which is used as the input of the RPN network. Nine anchor points with different area ratios and aspect ratios slide on the feature map to generate an RoI. The cerebral edema target in the image is very small. According to the total number of single cerebral edema pixels, RPN uses the SoftMax-Loss layer to train and classify the generated anchor points. The Smooth L_1_ layer was used to modify the anchor point coordinates to avoid gradient explosion problems^24^.

#### Target detection and instance segmentation (RoIAlign + FC/FCN)

The corresponding features of each necessary RoI are extracted. The RoI is extracted from the feature map and sent to the FC network for target classification, bounding-box regression, and instance segmentation. Before entering FC, RoIAlign was applied to adjust the size of each RoI to meet the input requirements of FC. Bilinear interpolation is used for RoIAlign to extract the corresponding features of each RoI on the feature map, which is replaced by the rounding operation of RoI pooling in Faster R-CNN^25^.

The model uses the multi-branch prediction network and the FC layer of classification prediction for the coordinate correction of the bounding box. The regression layer is used for instance segmentation. FCN is used to generate the target mask. In this study, the model parameter NUM_CLASSES was 2, which is the category and background area of cerebral edema. BACKBONE uses Resnet101’s residual network Road. LEARNING_RATE was 0.001. WEIGHT_DECAY was 0.0001.

After the above-mentioned network layer is given, the problems of vanishing gradient and exploding gradients are dealt with through nonlinear activation. Nonlinear functions have been discussed in many studies. Rectified linear units (ReLU) in this study is the most popular activation function. Equation (3) is described as:

3 $$ReLU\left(x\right)={\{}_{0, \quad otherwise.}^{x,\quad for \quad x>0}$$

ReLU is usually used in the activation function, because it is easy to calculate the process, make the network more diverse, alleviate the problem of overfitting, and promote efficient training of deep neural networks.

### Training the Mask R-CNN edema segmentation (MRES)

The pre-training weight Mask_rcnn_coco.h, based on the COCO data set, is introduced before model training. Transfer learning is used to solve the problem of a small training set^26^. COCO is a huge data set, which is used for object detection and image segmentation with 81 categories of images. Even if the training set is small, the parameters of the model can be adjusted to be better based on the pre-trained model. The ResNet101 residual network is used in this study, which has been constructed as the backbone network of Mask R-CNN. ResNet101 has the highest detection accuracy, which is combined with the FPN architecture to extract and segment the cerebral edema feature under study.

### Ethical statement

Institutional Review Board (IRB) approval was obtained from the Chang Gung Memorial Hospital IRB (approval number: 201900565B0), and the requirement for informed consent was waived given the retrospective nature of the study. All patients were not directly involved, the requirement for informed consent was waived by the same ethics committe.

## Results

The sample data of this study is an imaging study of cerebral edema complications caused by intracranial tumor patients undergoing stereotactic radiotherapy. Three algorithms are used to evaluate the correlation between each layer of cerebral edema image segmented by the algorithm and the volume described by the clinician’s analysis. Two-dimensional image processing is used to describe medical image preprocessing to solve the problems of insufficient image samples and unobvious features of cerebral radiation edema. In the deep learning model, there can be better enhancement results. Four image evaluation indicators are used to calculate the image volume of cerebral radiation edema and the standard ratio of the volume drawn by the clinician to verify the correlation between the two volumes.

This study was conducted under the deep learning development framework of tensorflow and Keras. Two segmentation methods were selected during the experiment: region growing and Mask R-CNN. These two methods were used as a comparison with the clinicians’ standard segmentation results. The specific results of this study are divided into two parts: (a) display the 2D image analysis of the two segmentation methods, and (b) perform evaluation index analysis on the segmentation results.

### Experiment and evaluation of edema

The following performance measures are utilized to evaluate the images of cerebral edema complications.

DICE index: For a given image set, the DICE index was used to measure the similarity between the area predicted by the model and its standard authenticity. The DICE index is given by Eq. (4).

4 $$DICE\, Index=\frac{2\left|\left(R\cap GTS\right)\right|}{\left|R\right|+\left|GTS\right|}$$

The evaluation equation can be used for the evaluation of volume similarity. It is an ensemble similarity measurement function and is usually used to calculate the similarity of two image samples.

Intersection over Union (IoU) index: For a given image set, IoU is used to measure the similarity between the area predicted by the model and its standard authenticity. IoU is given by Eq. (5)

5 $$IoU\, Index=\frac{\left|R\cap GTS\right|}{\left|R\cup GTS\right|}$$

This equation can be used for a similar evaluation of the image bounding box. The parameters R and GTS are used for segmented prediction and standard ground truth bounding boxes.

Volumetric overlap error (VOE) index: For a given image set, VOE is used to measure the similarity between the area predicted by the model and its standard authenticity. VOE is given by Eq. (6).

6 $$VOE\left(R, GTS\right)=1-\frac{\left|R\cap GTS\right|}{R\cup GTS}\times 100\%$$

Equation (6) is the intersection of the number of voxels in the pixel segmentation and the GTS, which is divided by the number of voxels. To evaluate the error-index of the two voxels, in the union of the segmented real volume and the reference volume, a VOE value of 0 represents a perfect segmentation ratio. The smaller the segmentation, the higher the similarity^27,28^.

### Experimental setup

The Mask R-CNN model in the open-source library is used as an open-source model. The pre-trained model was transferred to the COCO dataset, which was used for training learning. It is used to solve the problem of small data sets in medical imaging. Because many general features have been extracted, ResNet101 + FPN was used as the backbone network. Two methods, ResNet101 + FPN and region Growing algorithm, were used to analyze and evaluate with the standard images drawn by clinicians.

### 2D Experiment image analysis

**Figure 5:**
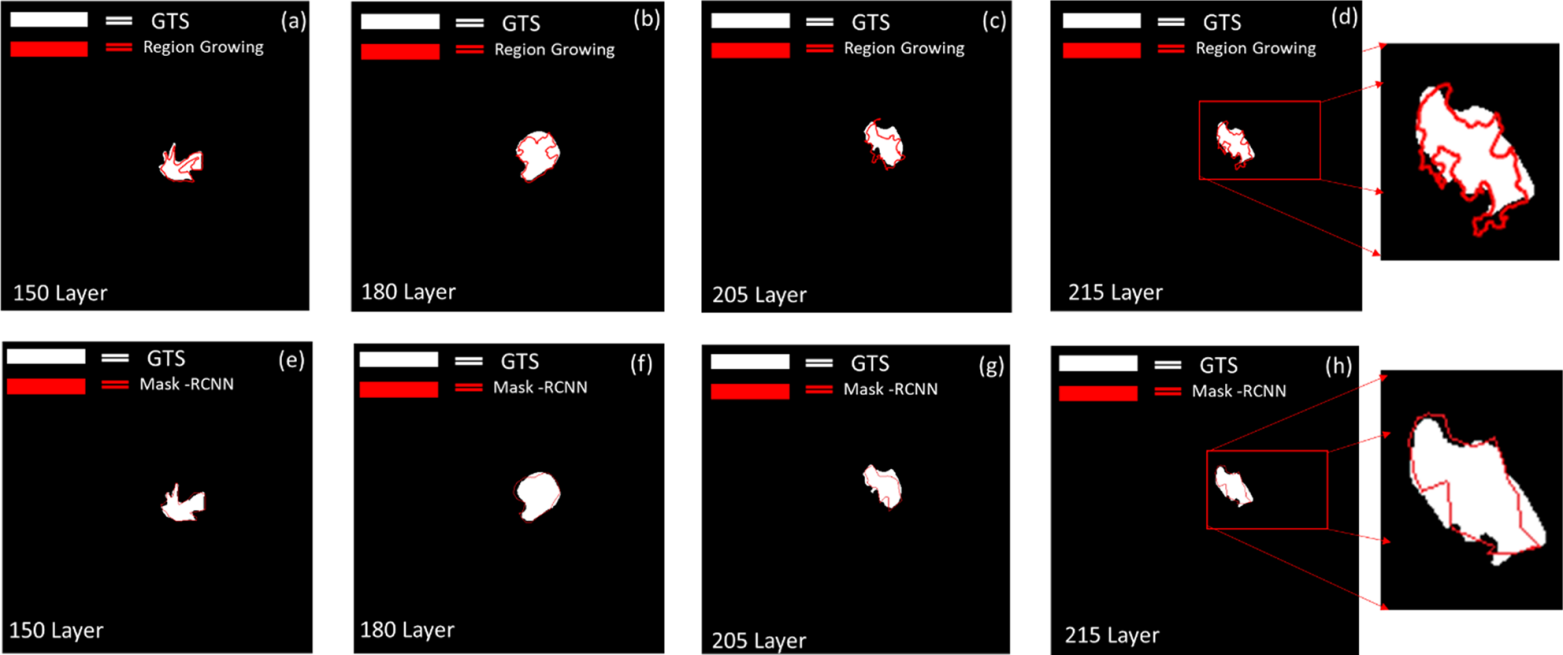
Cerebral edema Complications GTS image comparison between region growing and Mask R-CNN method. Panel (**a**–**d**) show the 2D segmentation result by Region growing method of cerebral edema complication image at 150, 205, 180, 215 layer. Panel (**e**–**h**) show the 2D segmentation result by Mask R-CNN model of cerebral edema complication image at 150, 205, 180, 215 layer. *GTS* ground truth segmentation, *R-CNN* region-based convolutional neural networks. Image source statement: This image was created using CyberKnife M6 system. Software URL: https://www.accuray.com/resources/accurays-cyberknife-m6-series/.

The results of using the region growing algorithm are shown in Fig. 5a–d. The set seeds were used to select the cerebral edema area. After the seed is set, the cerebral edema area was distinguished according to the pixel intensity around the cerebral edema image. The contour of cerebral edema can be segmented, as shown in Fig. 5, to depict the range of cerebral edema with a red contour. However, the segmentation effect in some areas was not so good.

The results of segmentation using the deep learning model are shown in Fig. 5e–h. The area contour of Mask is superimposed with the GTS described by the clinician. The two images, Fig. 5d,h, are compared with an enlarged size. The red curve segmented by Mask R-CNN deep learning is less likely to be over-segmented. This situation is closer to the result of GTS. Figure 5d of region growing has many regions that are segmented to the outside area. This situation will make the results of the evaluation indicators inaccurate. This study uses the above three-volume similarity evaluation indexes to analyze the segmentation results of these two methods and GTS.

**Figure 6:**
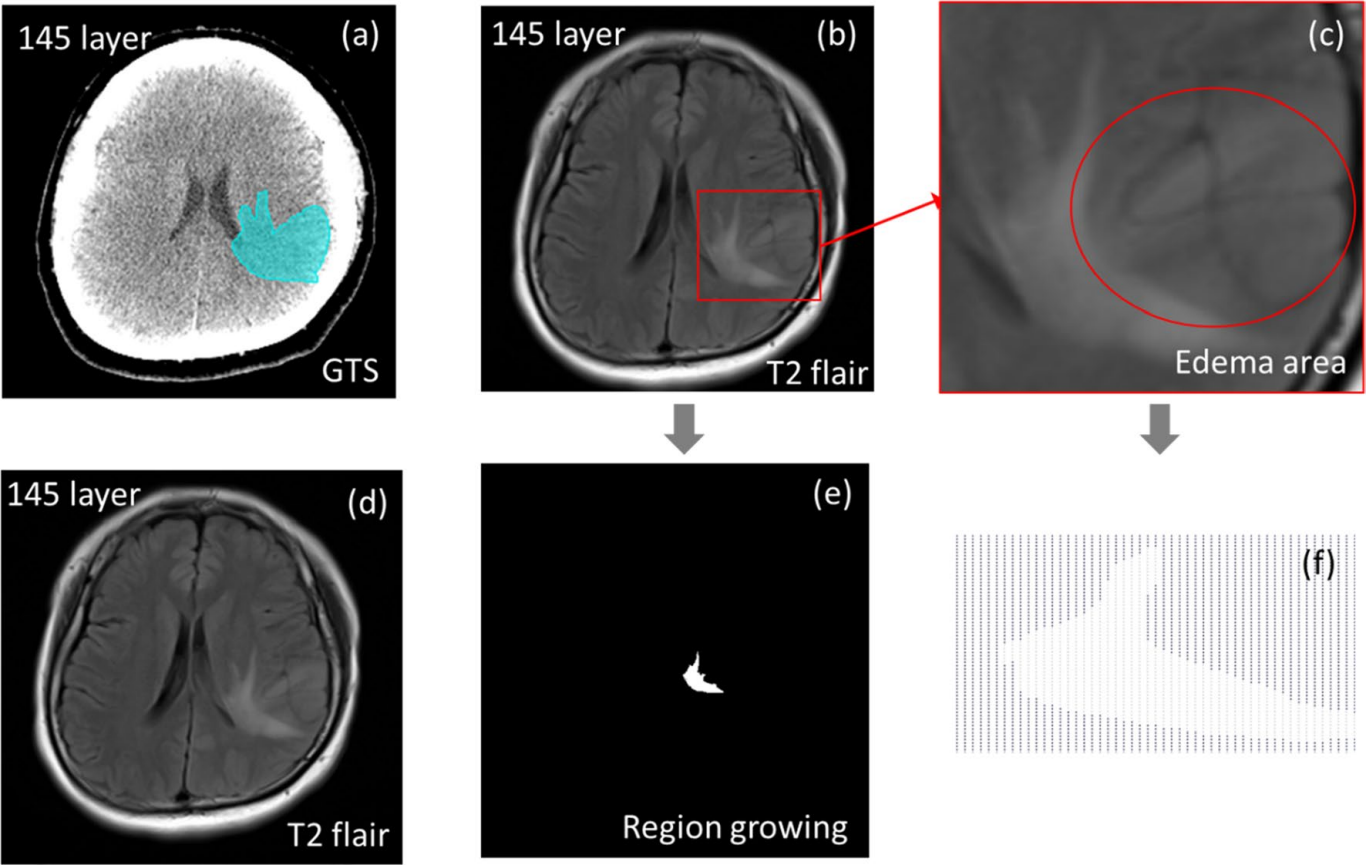
Cerebral edema Complications image comparisons between T2 flair image with region growing method and GTS image. Panel (**a**) shows the GTS image at 145 layer. Panel (**b**) shows the T2 flair image with region of interest. Panel (**c**) shows the edema area that boundary strength is not obvious. Panel (**d**) shows the T2 flair image. Panel (**e**) shows the segmentation result by using region growing method. Panel (**f**) shows the edema area which boundary strength is not obvious display by regional growth algorithm features. Abbreviations: GTS: ground truth segmentation. Image source statement: This image was created using CyberKnife M6 system. Software URL: https://www.accuray.com/resources/accurays-cyberknife-m6-series/.

For each layer of cerebral edema, the model uses pre-set seeds to give segmentation instructions. The segmentation results of the region growing algorithm are shown in Fig. 6.

It can be seen that the segmentation results of the region growing algorithm are different from the cerebral edema complications described by the clinician. The reason is that clinicians will draw a larger outline based on whether the damaged cells around the area of cerebral edema complications need to be evaluated, which is shown in Fig. 6a. Figure 6b,c show a cerebral edema with not strong intensity. The segmentation of the region growing algorithm is shown in Fig. 6d, which will segment the area according to the intensity around the cerebral edema complication region and will not over segment other regions with different intensity, so the boundary of some regions cannot be segmented.

### Segmentation result evaluation index analysis

**Figure 7:**
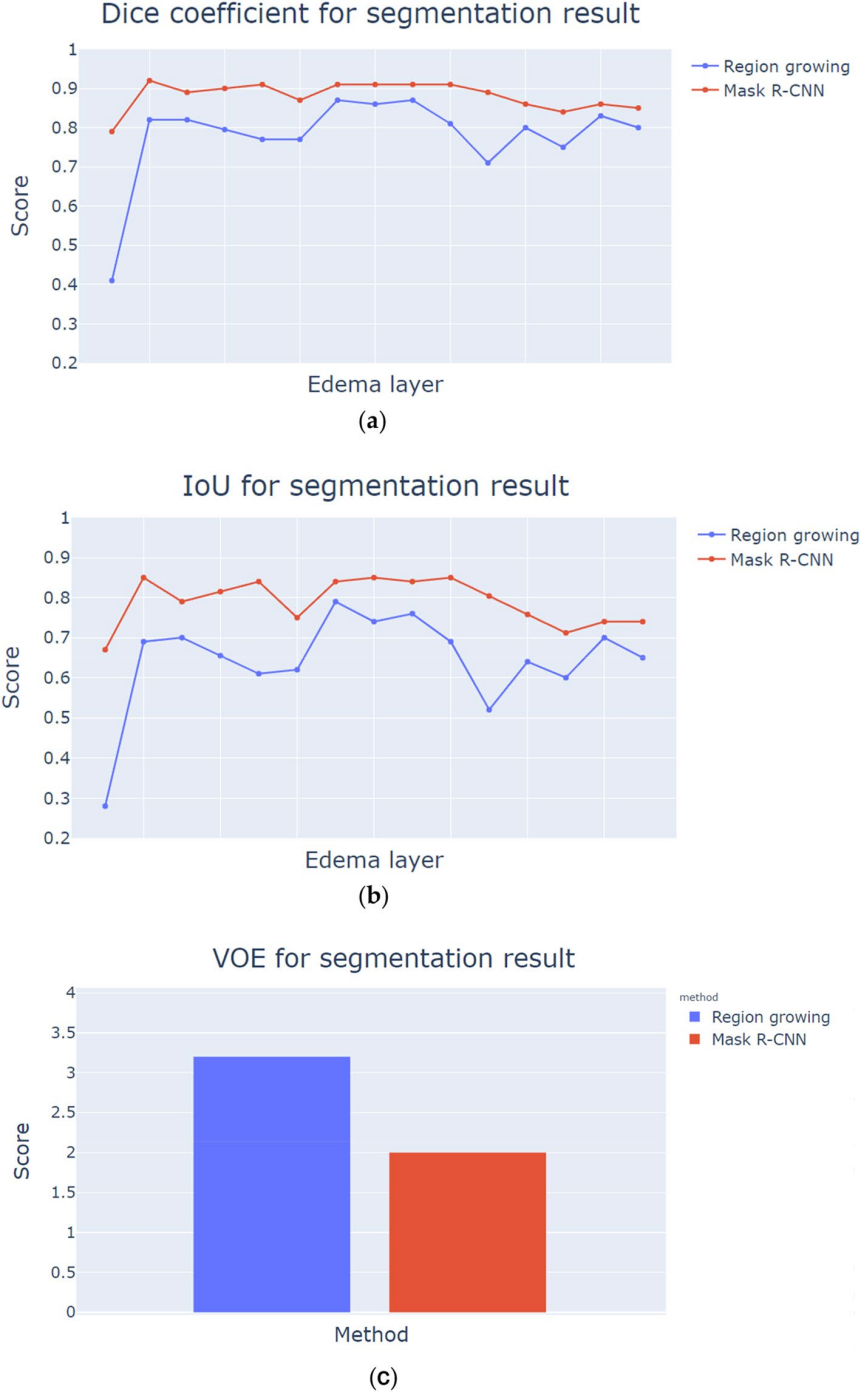
Evaluation index for segmentation results. (**a**) DICE index, (**b**) IoU index, (**c**) VOE index. *R-CNN* region-based convolutional neural networks, *IoU* intersection over union, *VOE* volumetric overlap error. Image source statement: This image was created using the plotly library of Python 3.7. Software URL: https://www.python.org/.

The analysis of the correlation between the volume of the algorithm segmentation result and the volume of the cerebral radiation edema, Eqs. (4) and (5), is imported as an indicator and calculating with each layer of the cerebral edema image. The value of the DICE index and the value of IoU were close to 1, which means that the segmented volume of the algorithm has a better correlation with the standard radioactive cerebral edema volume. This correlation also means that the result is closer to the range outlined by the clinician, as shown in Fig. 7.

The effect of Mask R-CNN and region growing with applying deep learning were as follows: the DICE index was 0.88, and the IoU index was 0.79 using Mask R-CNN. The DICE, IoU, and VOE indices using region growing were 0.77, 0.64, and 3.2, respectively. Mask R-CNN had the best segmentation effect of the two algorithms. For the VOE index in Eq. (6), the algorithm segmentation index ratio was close to 0 and had a better correlation. In Fig. 7c, the effect of the VOE evaluation index with Mask R-CNN deep learning segmentation was 2.0, which was the best segmentation of the two algorithms.

## Discussion

In this study, the accuracy of segmentation of cerebral edema complications using the Mask R-CNN model was closer to the standard similarity described by clinicians. The vast majority of current clinical and research work to evaluate complication imaging must rely on physician experience. The segmentation algorithm based on Mask R-CNN can represent a method to reduce subjectivity and provide accurate analysis to assist clinical decision-making and improve the time spent for physicians to depict images of complications of cerebral edema.

Although the region growing method can be simply expressed as the classification of cerebral edema pixel areas, in this study, clinicians considered that the surrounding cells of the complications might also have focal areas. Therefore, the oncologists described the area wider, which causes inaccurate segmentation. Nowadays, various automatic and semi-automatic organ segmentation algorithms have been proposed. A fully automatic deep learning segmentation method was used for cerebral edema complications caused by radiotherapy in patients with intracranial tumors in this study. The segmented cerebral edema images and clinical standard segmentation results were analyzed.

The samples of this study are drawn from clinical radiation oncologists. Currently, there are only a few clinical cerebral edema data sets. Although this study highlights the advantages of using Mask R-CNN on a small data set, the data set can be expanded in future studies to improve more accurate and stable predictions. However, it is a challenge to establish a large number of complication image data sets. Large amounts of data require a lot of time for physicians to label. Labeling standards should also be unified. This will increase the difficulty of labeling, which is a limitation in AI technology.

Deep learning neural networks usually learn by using backpropagation of loss. To perform deep learning network learning, the model needs to train an image data set. Label images are added to each database through a marker. At present, there are many methods for automatic labeling image acquisition in deep learning networks for labeling images. A method proposed by Kye-hyeon et al. optimizes the sampling process of manual labeling and reduces the labor cost of labeling^29^.

When the Mask R-CNN model was established in our research, the label image of the cerebral edema image was input into computer training based on the results drawn by clinicians as a reference. Because this step is very cumbersome and time-consuming, in the future, auto labeling of cerebral edema imaging regions will be achieved. The most suitable cerebral edema area in the clinical medical image was found. Then, we recorded the value of the cerebral edema area for the computer to judge and learn. In the future, when clinicians are marking cerebral edema images, they only need to modify the image to outline the non-cerebral edema area, which can effectively reduce the labeling time and obtain a large number of effective training data sets.

The establishment of a supervised model depends on the quality of preprocessing or post-processing^30^. When the input image data is unevenly distributed, or features are not obvious, the performance of the supervised segmentation method is often very poor. Adrian V. Dalca et al. proposed a principle method for unsupervised segmentation. This method is used to train CNN on a dataset without any manually annotated images, which proves that the proposed method can achieve an unsupervised response in different MRI images with the segmentation of brain MRI images^31,32^.

When the number of manually segmented images is limited, Pawel Mlynarski et al. used a weakly supervised method to annotate images. Compared with standard supervised learning, this method can significantly improve the segmentation effect. The observed improvements are related to weakly annotated images that can be used for training^33^.

Mask R-CNN was originally developed for object detection and object instance segmentation of natural images. Jeremiah et al. used Mask R-CNN, which can be used to perform efficient automatic segmentation of various cells acquired under various conditions and widely segment the microscope image of the nucleus. In addition, it has been shown that the cyclic learning rate mechanism allows effective training of Mask R-CNN models without fine-tuning the learning rate, thereby eliminating the manual and time-consuming aspects of the training process^34^.

In the application research of Rajaram Anantharaman et al. in the field of oral pathology to segment cold sores and canker sores, there are few public data sets on ulcers and cold sores. Although these studies emphasize the advantages of using Mask R-CNN on small data sets, future research will focus on expanding the size of the training data to account for more image changes. Therefore, when this study adds more data in the future, it will rely on the importance of automatic labeling, which can reduce the time described for clinicians^35–37^.

Murali et al. explored the use of two methods to segment brain tumors. The first method uses a combination of WNet and UNet to segment brain tumors. The second method uses the Mask R-CNN framework to classify tumors in the brain^38^. It was proven that Mask R-CNN could be used to efficiently and automatically segment microscopic images for various types of images. Therefore, this study refers to a variety of information on the segmentation of medical images and adopts Mask R-CNN.

Maraka et al. studied brain edema in patients with brain tumors caused by laser interstitial thermal therapy (LITT) combined with radiotherapy^39^. The study cohort included eight patients: six with glioblastoma, one with anaplastic astrocytoma, and one with metastases. MRI showed that cerebral edema worsened in one patient after radiotherapy. Their conclusions indicate that continuous treatment with LITT and RT can cause cerebral edema. Although the treatment time may be prolonged, it can usually be successfully controlled with steroids. However, we use more cases and explain more extensively the complications of cerebral edema caused by radiotherapy in patients with Intracranial tumors.

Dong et al. used a U-Net-based fully convolutional networks to automatically detect and segment brain tumors^40^. In their research, due to GPU memory limitations, the 2D U-Net model can process complete slices at one time, while the 3D convolution system can only process small blocks that cover a small part of the 3D volume. Therefore, a 2D-based network is used. In addition, they proposed a fully automated brain tumor segmentation method, which was developed using a deep convolutional network based on U-Net. Their method was evaluated on Multimodal Brain Tumor Image Segmentation (BRATS 2015) dataset. The results of cross-validation show that the method can effectively obtain promising segmentation. Compared with our study, this result verifies that similar methods have the same excellent results. We still hope to have more cases in future studies, so that the explanation of the complications of cerebral edema caused by radiotherapy in patients with intracranial tumors can be more clear.

## Conclusions

Most of the clinical evaluation of cerebral edema complications images must rely on the experience of clinical and research physicians. The description of medical images is time-consuming. The interpretations of each physician are inconsistent. The method of this research effectively improves the efficiency and consistency of the accuracy of this work. This study analyzes the imaging conditions of intracranial tumor patients following radiation therapy after four months of treatment. Currently, there is no image outline study of cerebral edema complications, so we used two algorithms for automatic outline segmentation on medical images, namely the region growing method for outlining clinical images and the Mask R-CNN model used in this study. The Mask R-CNN model based on deep learning is the closest to the standard outlined by clinicians for cerebral edema complications, which is the best result. It also provides accurate and stable segmentation predictions. From the results of this study, the Mask R-CNN model has great advantages over the segmentation algorithm model used in clinical practice. It also reduces subjectivity, provides accurate analysis to assist clinical decision-making, and saves the time for physicians to describe medical images of cerebral edema complications. In the current traditional method, the clinician will consider that the surrounding cells of the complication may still have a focus area, so the clinician will describe the area very conservatively, resulting in a wider segmentation area and inaccurate situation. Various automatic and semi-automatic organ segmentation algorithms have been proposed in the existing research. In this study, we applied a fully automated deep learning segmentation method to intracranial complications cerebral edema and analyzed the segmented cerebral edema images and clinical standard results. Therefore, it can be applied to clinical work in the future to achieve good complication segmentation. It can also provide clinical evaluation, guidance, and advice, and assist the clinic in the description of RoI in the future. Clinical radiation oncologists described the samples of this study. There are still a few clinical cerebral edema data sets. However, this study highlights the advantages of using Mask R-CNN on small data sets. Future research still needs to expand the training data set to improve more accurate and stable predictions. When building the Mask R-CNN model, the cerebral edema images are labeled based on the results drawn by the clinician. However, this step is very tedious and time-consuming. Therefore, in the future, efforts can be made to automatically label the cerebral edema image area. When annotating images, clinicians only need to correct the image to outline the non-cerebral edema area. It can effectively reduce the annotation time and obtain a large number of effective training data sets.
